# Variation in the recall of socially rewarding information and symptoms of generalised anxiety: evidence from two cohorts

**DOI:** 10.1186/s12888-025-07402-1

**Published:** 2025-10-21

**Authors:** Chi Ling Yeung, Peiyao Tang, Gemma Lewis, Glyn Lewis, Nicola Wiles, Jessica K. Bone

**Affiliations:** 1 Division of Psychiatry, University College London, London, UK; 2 Centre for Academic Mental Health, Population Health Sciences, Bristol Medical School, University of Bristol, Bristol, UK; 3 Research Department of Behavioural Science and Health, Institute of Epidemiology & Health Care, University College London, London, UK

**Keywords:** Generalised anxiety disorder, Memory, Cohort study, RCT

## Abstract

**Background:**

Cognitive theories suggest that anxiety symptoms are associated with increased recall of threatening information, but previous evidence has been inconsistent. We examined whether recall of socially rewarding or threatening information was associated with concurrent and subsequent generalised anxiety disorder (GAD) symptoms.

**Methods:**

We used data from a cohort study (*N* = 530, 68% female) and the baseline of a randomised controlled trial (*N* = 653, 58% female). All participants had a history of depressive symptoms. Both studies included a computerised task assessing incidental word recall and measured GAD symptoms using the Generalised Anxiety Disorder Assessment (GAD-7). We tested concurrent associations in both samples and lagged associations in the cohort, which measured GAD scores at four time-points (two weeks apart) and recall at the first three time-points. We used multilevel linear (cohort) and linear (RCT) regression models, before and after adjusting for confounders.

**Results:**

In the cohort, there was strong evidence that better recall of socially rewarding information was associated with lower GAD scores concurrently (coef=-0.18; 95% CI=-0.31–0.04). There was no evidence for an association with recall of socially threatening words (coef=-0.04, 95% CI=-0.20-0.12). Longitudinally, there was no evidence that recall of socially rewarding or threatening information was associated with subsequent GAD scores. In the RCT, there was evidence that better recall of socially rewarding information was associated with lower concurrent GAD scores (coef=-0.32; 95% CI=-0.56–0.08).

**Conclusions:**

GAD may be characterised by difficulty in recalling socially rewarding information but not memory for socially threatening information. Our findings indicate that recalling less socially rewarding information may be a marker of current GAD symptoms, but not a risk factor longitudinally.

**Supplementary Information:**

The online version contains supplementary material available at 10.1186/s12888-025-07402-1.

## Introduction

Generalised anxiety disorder (GAD) is characterised by persistent, excessive and uncontrollable worry about multiple domains in life. It is the most common mental health issue, with a large global burden of disease [1]. Cognitive theories of anxiety suggest that bias toward potentially threatening information plays a role in the aetiology of GAD [2–4]. There is strong evidence for cognitive bias in both attention and interpretation among people with GAD. For example, individuals with GAD are more likely to attend to threatening information and may be more likely to interpret ambiguous information as threatening [4–9].

Evidence on recall of threatening information in people with GAD is less consistent than the evidence on attention and interpretation. Several meta-analyses have provided evidence that, compared to less anxious individuals, people with higher levels of anxiety have good recall of negative material [10] and poor recall of positive material [11]. This bias in memory for threatening information was also found to be present across different anxiety disorders. People with GAD, panic disorder, social phobia and post-traumatic stress disorder all had better recall of threatening stimuli compared to healthy control groups [11]. However, previous evidence for the association between recall of threatening information and GAD has been mixed. In one study, better recall of threatening information was found in individuals with GAD compared to the non-anxious group [12]. In contrast, another study found poorer recall of threatening words in individuals with higher compared to lower levels of anxiety [13]. Additionally, several other cross-sectional studies found no evidence for differences in the recall of threatening information in individuals with GAD compared to those without [14–18].

The lack of consistent evidence for associations between recall of socially rewarding and threatening information and GAD could be due to the methodological limitations of existing studies. Most studies were small, with limited statistical power (*n* < 55). Studies have generally been observational and have used case-control designs [13, 14, 16, 18], which are more susceptible to selection bias than cohort and cross-sectional studies, unless controls are selected from the same population as the cases [19]. By dichotomising participants into anxious and control groups, previous studies have been unable to examine biases in recall across the whole continuum of anxious symptom severity. Another limitation is the lack of longitudinal studies to establish temporality in the relationship between recall bias and GAD and rule out reverse causation. This means we do not know whether changes in recall bias precede changes in GAD symptoms, and thus potentially have a causal role in the development of GAD, or instead changes in GAD symptoms lead to changes in recall bias. While a few longitudinal studies have tested the association between recall of positive and negative information and depression [20–22], there has been no longitudinal study of GAD and positive and negative information processing to our knowledge. Exploring whether changes in recall precede changes in anxiety symptoms would improve our understanding of the cognitive aetiology of anxiety symptoms.

In this study, we investigated the cross-sectional and longitudinal associations between recall of socially rewarding and socially threatening information and GAD symptom severity. We used data from two large samples, a prospective cohort study and a randomised controlled trial. We examined associations between recall of socially rewarding (positive) and socially threatening (negative) information and GAD symptom severity, measured concurrently and two weeks later. We hypothesised that better recall of socially threatening information would be associated with more severe GAD symptoms, and better recall of socially rewarding information would be associated with less severe GAD symptoms.

## Methods

### Design

We conducted secondary analyses of data from the PANDA research programme; indications for Prescribing ANtiDepressAnts (PANDA) that lead to a clinical benefit [23]. We used data from the PANDA cohort study (cohort) and the PANDA pragmatic double-blind placebo-controlled randomised trial (RCT; [23–25]), The cohort study and RCT both recruited individuals experiencing depressive symptoms from UK primary care surgeries and included the same recall task and measures of GAD symptoms. Both studies were led by GlL and GeL. We examined associations between recall bias and measures of GAD symptoms in both samples in this study.

The original aim of the PANDA cohort study was to estimate a clinically important difference on commonly used self-administrated questionnaires for depressive symptoms [26]. The cohort had four time-points, each two weeks apart, with a total follow-up period of six weeks. We used data from all four time-points. Interviews were conducted at participants’ homes or GP surgeries.

The PANDA RCT investigated the clinical effectiveness of sertraline in UK primary care and tested how depression severity and duration affected treatment response [24]. We used data from the baseline interview, which was conducted before randomisation at participants’ homes, GP surgeries or universities. Baseline data from the RCT therefore provides an unmedicated replication sample, allowing comparison across two cohorts with and without antidepressant use, which together provide a good representation of people in primary care.

### Participants

#### PANDA cohort

Participants aged 18 to 70 were recruited from General Practice (GP) surgeries in three UK sites (Bristol, Liverpool and York). Participants who had reported depressive episodes, depressive mood or depressive symptoms (of any severity or duration) in the past year were identified by searching computerised records at each site. There were no restrictions on whether individuals were taking antidepressants nor had been referred to psychological therapy services. Individuals were excluded if they had a diagnosis of bipolar disorder, psychosis or an eating disorder; had alcohol or substance use problems; were unable to complete study questionnaires; or were pregnant at 30 weeks or more.

Eligible individuals (*N* = 7721) were sent study invitations by post and 1470 (19%) replied. Of these, 821 (55%) were willing to be contacted, 23 (3%) of whom were ineligible. The remaining 798 were contacted to arrange an interview and 563 (71%) consented. At baseline, 558 participants provided data (5 could not be contacted); 476 (85%), 443 (79%) and 430 (77%) participants provided data at two, four, and six weeks respectively. The final analytical sample for concurrent analyses consisted of 530 participants, who completed the recall task and GAD measure at one or more time-points. For lagged analyses, the final analytical sample consisted of 460 participants, who completed the recall task and GAD measure at two or more time-points. There were no significant differences in the demographics and recall task performance of participants who dropped out (i.e., completed three or fewer time-points) compared to those who completed all four time-points (Table S1).

Ethical approval was obtained from NRES Committee South West — Central Bristol and all participants provided written informed consent.

#### PANDA RCT

Individuals aged 18 to 74 years were eligible if they had sought treatment for depressive symptoms (of any severity or duration) in the past two years and there was clinical uncertainty about the benefit of taking antidepressants. Participants were recruited from 179 primary care surgeries across four UK cities (Bristol, Liverpool, London, and York). They were either identified by GPs during a consultation or sent an invitation following a database search. Individuals were excluded if they had: used antidepressants in the past 8 weeks; comorbid psychosis, schizophrenia, mania, hypomania, bipolar disorder, dementia, eating disorder, or major alcohol or substance abuse; or medical contraindications for sertraline.

From January 2015 to August 2017, 31,645 eligible individuals were sent invitations to the study by post and 4428 (14%) replied. Of these, 1029 (23%) were willing to be contacted and 893 (87%) were eligible according to their GP. Another 427 patients were identified at GP consultation. In total, 1320 individuals were checked for eligibility, 671 (51%) were eligible. Of these, 655 (98%) gave consent, although 2 (0.3%) were excluded due to missing baseline assessments. Therefore, at baseline, 653 (97%) participants were interviewed before randomisation to sertraline or placebo, and were included in this study.

Ethical approval was obtained from the National Research Ethics Service Committee, East of England — Cambridge South and all participants provided written informed consent.

### Measures

#### Anxiety symptoms

The Generalised Anxiety Disorder Assessment (GAD-7; [27]) is a seven-item self-report measure of GAD symptoms during the past two weeks. Total scores range from 0 to 21, with higher scores indicating more severe symptoms. The GAD-7 demonstrated high internal consistency in both the cohort sample (Cronbach’s alpha range 0.89–0.93 across time-points) and the RCT sample (Cronbach’s alpha = 0.87 at baseline). The GAD-7 was administered at all time-points in the cohort and RCT.

#### Diagnoses of GAD and depression

The self-administrated Clinical Interview Schedule-Revised (CIS-R; [28]) was used to assess depression or GAD according to International Classification of Diseases (ICD-10) criteria. Participants completed the CIS-R at baseline in the cohort and RCT.

#### Incidental recall task

A computerised task tested incidental recall of socially rewarding and threatening information. Twenty socially rewarding (positive, e.g., cheerful, capable and honest) and 20 socially threatening (negative, e.g., hostile, untidy and neglectful) personality characteristics were presented on a computer screen in random order, each for 500 milliseconds. Participants were asked to indicate whether they would like or dislike overhearing someone describing them in this way by pressing a key on the keyboard. Immediately after the task, participants were given two minutes to recall as many of these words as possible.

Socially rewarding and threatening words were paired and matched in length, frequency of use and meaningfulness. The numbers of socially rewarding and threatening words accurately recalled were computed as positive and negative hits respectively (total possible ranges 0–20). In the cohort study, the task was administered at baseline and two- and four-week follow-up assessments (time-points one to three). In the RCT, the task was administered at baseline and two- and six-week follow-up, but only the baseline administration was used in this study (before randomisation to sertraline or placebo). The recall element of this task was unexpected at baseline but may have been anticipated by participants at time-points two and three. A different set of words was presented at each time-point (120 words in total) with the order randomised across participants.

#### Confounders

Potential confounders were baseline age (under 50 years, 50 or above), gender (male, female), highest level of education (GCSE or below, A-level or above), negative life events, and antidepressant use. Negative life events in the past six months were self-reported and categorised using a binary variable (none, one or more). In the cohort study, current use of antidepressants was assessed by self-report at all time-points and recorded as a binary variable (yes, no). In the RCT, past antidepressant use was reported at baseline (yes, no).

### Statistical analyses

Analyses were performed using Stata 16 [29]. We report descriptive data for categorical variables using numbers and percentages and continuous variables using means and standard deviations.

#### Concurrent associations

In the cohort, multilevel linear models were used to test concurrent associations between positive and negative hits (continuous exposures) and GAD scores (continuous outcome). We used data from all available time-points (one, two, and three; the recall task was not included in the study at time-point four). All models had a random intercept for participant to allow for the clustering of responses within individuals over time-points and a fixed effect for time ([30]; time coded as a categorical variable). In the RCT, linear regression was used to test the concurrent association between positive and negative hits (continuous exposures) and GAD scores (continuous outcome) at baseline. All models are presented before and after adjustment for confounders, and positive and negative hits were included as exposures in the same model. Participants were included in analyses if they had complete data at one or more time-point (listwise deletion was used to handle missing data).

#### Lagged associations

In the cohort, we used multilevel linear models to test lagged associations between positive and negative hits at time-points one to three (exposure) and GAD scores at time-points two to four (outcome). After first adjusting for potential confounders, we then additionally adjusted for baseline GAD score. The fully adjusted lagged models thus test the association between positive and negative hits and GAD score two weeks later after accounting for previous GAD scores. Participants were included in analyses if they had complete data at two or more time-points (listwise deletion was used to handle missing data).

#### Sensitivity analysis

In a sensitivity analysis, we combined positive and negative hits into the total number of hits. We used total hits as a continuous outcome, testing associations with word valence (positive/negative; binary exposure) and GAD scores (continuous exposure). We also included an interaction between word valence and GAD scores on total hits. This allowed us to test whether the association between positive hits and GAD scores differed significantly from the association between negative hits and GAD scores. Where there was evidence for an interaction, we tested associations between GAD scores and hits separately for positive and negative words. Multilevel linear models were used in the cohort sample (using data from time-points one to three) and linear regressions in the RCT.

## Results

The cohort included 530 participants (68% female) aged 18–71 years (mean = 48.2, standard deviation [SD] = 12.6). At baseline, GAD score ranged from 0 to 21 (mean = 7.20, SD = 5.58) and 43% of participants met diagnostic criteria for GAD on the CIS-R (Table 1). Participants made a mean of 2.39 (SD = 1.80) positive hits and 1.65 (SD = 1.40) negative hits.

**Table 1:** Demographic and clinical characteristics and recall performance according to these characteristics for each sample at baseline

		Cohort (*N* = 530)			RCT (*N* = 653)		
		*N* (%)	Positive hits	Negative hits	*N* (%)	Positive hits	Negative hits
			Mean (SD)		Mean (SD)		
Age	Under 50	278 (53%)	2.83 (1.89)	2.00 (1.47)	469 (72%)	3.06 (1.75)	2.22 (2.79)
	50 or older	252 (48%)	1.91 (1.56)	1.25 (1.20)	184 (28%)	2.57 (1.68)	1.70 (1.31)
Gender	Male	169 (32%)	2.06 (1.61)	1.33 (1.24)	265 (41%)	2.86 (1.83)	1.77 (1.42)
	Female	361 (68%)	2.55 (1.86)	1.80 (1.45)	387 (58%)	2.97 (1.69)	2.27 (2.98)
Ethnicity	White	513 (97%)	2.36 (1.74)	1.62 (1.35)	579 (89%)	2.96(1.75)	2.09 (2.57)
	Non-white	17 (3%)	3.59 (2.90)	2.53 (2.35)	73 (11%)	2.68 (1.67)	1.97 (1.56)
Education level	GCSE or below	202 (38%)	1.88 (1.61)	1.22 (1.20)	203 (31%)	2.23 (1.48)	1.42 (1.28)
	A level or above	328 (62%)	2.71 (1.84)	1.91 (1.45)	450 (69%)	3.24 (1.76)	2.36 (2.81)
Marital status	Married or cohabiting	271 (51%)	2.35 (1.81)	1.67 (1.40)	255 (39%)	2.84 (1.78)	2.08 (3.51)
	Single, separated, divorced or widowed	259 (49%)	2.45 (1.79)	1.63 (1.40)	398 (61%)	2.99 (1.72)	2.07 (1.49)
Employment status	Employed	290 (55%)	2.58 (1.82)	1.84 (1.47)	437 (67%)	3.02 (1.71)	2.16 (2.82)
	Unemployed	112 (21%)	1.93 (1.55)	1.49 (1.33)	73 (11%)	2.12 (1.35)	1.39 (1.30)
	Student	9 (2%)	3.33 (2.35)	2.11 (1.05)	68 (11%)	3.66 (1.91)	2.76 (1.77)
	Retired/full-time carer	119 (22%)	2.32 (1.85)	1.27 (1.21)	74 (11%)	2.46 (1.78)	1.61 (1.26)
Negative life events	None	175 (33%)	2.28 (1.72)	1.69 (1.42)	216 (33%)	2.92 (1.73)	2.01 (1.43)
	One or more	355 (67%)	2.45 (1.84)	1.63 (1.39)	436 (67%)	2.94 (1.75)	2.11 (2.87)
Antidepressant use ^a^		364 (69%)	2.40 (1.86)	1.63 (1.44)	391 (60%)	2.80 (1.71)	1.94 (1.40)
GAD diagnosis		227 (43%)	2.20 (1.75)	1.65 (1.49)	353 (54%)	2.95 (1.80)	2.20 (3.36)
Depression diagnosis		240 (45%)	2.21 (1.79)	1.59 (1.46)	355 (54%)	2.89 (1.71)	2.00 (1.41)
Comorbid GAD and depression		157 (30%)	2.12 (1.71)	1.55 (1.51)	-	-	-

*SD* standard deviation

Baseline recall data was available for 638 RCT participants (98%)

^a^Antidepressant use refers to current use in the cohort and past use in the RCT. In the cohort, 5 (1%) participants were missing data on antidepressant use. In the RCT, 1 participant (< 1%) was missing data on each of gender, ethnicity, antidepressant use, GAD diagnosis, depression diagnosis and employment status

The RCT included 653 participants (58% female) aged 18–73 years (mean = 39.7; SD = 14.9). At baseline, GAD score ranged from 0 to 21 (mean = 9.43; SD = 5.27), and 54% of participants met diagnostic criteria for GAD on the CIS-R. Participants made a mean of 2.93 (SD = 1.74) positive hits and 2.07 (SD = 2.48) negative hits.

### Concurrent associations

In the cohort, there was strong evidence that participants with more positive hits had lower GAD scores. Each additional hit was associated with a 0.18 point lower GAD-7 score (95% confidence interval [CI]=−0.31 to −0.04, *p* = 0.009, adjusted for confounders; Table 2). There was no evidence for an association between negative hits and GAD scores, before or after adjustment for confounders (coefficient [coef]=−0.04, 95% CI=−0.20 to 0.12, *p* = 0.62, adjusted for confounders).

**Table 2:** Concurrent associations between positive and negative hits (exposures) and generalised anxiety symptoms (GAD-7 score; outcome)

		Model 1: unadjusted				Model 2: adjusted^a^			
		*N*	Coef	95% CI	*p-*value	*N*	Coef	95% CI	*p-*value
Cohort	Positive hits	521	−0.17	−0.30 to −0.04	0.012	521	−0.18	−0.31 to −0.04	0.009
	Negative hits		−0.05	−0.21 to 0.11	0.516		−0.04	−0.20 to 0.12	0.616
RCT	Positive hits	639	−0.24	−0.48 to 0.00	0.053	637	−0.32	−0.56 to −0.08	0.010
	Negative hits		−0.01	−0.17 to 0.17	0.973		−0.05	−0.22 to 0.11	0.528

In each sample, positive and negative hits were both included in the same model. Cohort models used data from time-points one to three. RCT models used baseline data only

^a^Confounders were age, gender, education level, negative life events and antidepressant use (current in the cohort and past in the RCT)

In the RCT, there was also strong evidence that participants who made more positive hits had lower GAD scores (Table 2). For every positive hit, GAD-7 score decreased by 0.32 points (95% CI=−0.56 to −0.08, *p* = 0.010, adjusted for confounders). As in the cohort sample, there was no evidence for an association between negative hits and GAD scores, before and after adjustment for confounders (coef=−0.05, 95% CI=−0.22 to 0.11, *p* = 0.53, adjusted for confounders).

### Lagged associations

In the cohort, there was no evidence of a lagged association between positive hits and subsequent GAD scores (coef=−0.10, 95% CI=−0.24 to 0.03, *p* = 0.13, adjusted for confounders; Table 3). Additionally adjusting for baseline GAD scores did not materially alter the findings (coef=−0.05, 95% CI=−0.17 to 0.07; *p* = 0.44). There was also no evidence for an association between negative hits and subsequent GAD scores, before and after adjustment for baseline GAD scores (coef = 0.07, 95% CI=−0.08 to 0.22, *p* = 0.38, fully adjusted).

**Table 3:** Lagged associations between positive and negative hits (continuous exposures) at time-points one to three and subsequent anxiety symptoms (continuous outcome) at time-points two to four in the cohort

	Model 1: Unadjusted				Model 2: Adjusted^a^				Model 3: Additionally adjusted^b^			
	*N*	Coef	95% CI	*p-*value	N	Coef	95% CI	*p-*value	*N*	Coef	95% CI	*p-*value
Positive hits	460	−0.11	−0.24 to 0.02	0.106	460	−0.10	−0.24 to 0.03	0.127	456	−0.05	−0.17 to 0.07	0.435
Negative hits		−0.02	−0.17 to 0.14	0.843		−0.003	−0.16 to 0.16	0.972		0.07	−0.08 to 0.22	0.379

Positive and negative hits were both included in the same model

^a^Confounders were age, gender, education level, negative life events and current antidepressant use

^b^Additionally adjusted for baseline GAD-7 score

### Sensitivity analysis

We next tested models with total number of hits as the outcome, and GAD scores and word valence (positive/negative) as exposures (Table 4). In the cohort, there was strong evidence that participants made an average of 0.73 (95% CI = 0.64 to 0.82, *p* < 0.001 adjusted for confounders) more positive than negative hits. For every one-point increase in GAD-7 score, participants made 0.02 fewer hits (95% CI=−0.03 to 0.00, *p* = 0.009 adjusted for confounders). There was also strong evidence for an interaction between word valence and GAD scores on total hits, before (interaction term *p* = 0.003) and after (interaction term *p* = 0.002) adjusting for confounders. Testing associations between GAD scores and hits separately for positive and negative words, there was a stronger association between GAD scores and total hits for positive words (coef=−0.03, 95% CI=−0.05 to −0.01) than for negative words (coef=−0.01, 95% CI=−0.02 to 0.01).

**Table 4:** Concurrent associations from sensitivity analyses using total hits as a continuous outcome, testing associations with word Valence (positive/negative; binary exposure) and GAD symptoms (continuous exposure)

		Model 1: Unadjusted				Model 2: Adjusted^a^			
		*N*	Coef	95% CI	*p-*value	*N*	Coef	95% CI	*p-*value
**Cohort**	Valence	530	0.73	0.64 to 0.83	< 0.001	528	0.73	0.64 to 0.82	< 0.001
	GAD-7 score		−0.02	−0.03 to 0.00	0.022		−0.02	−0.03 to 0.00	0.009
**RCT**	Valence	639	0.86	0.62 to 1.09	< 0.001	637	0.86	0.63 to 1.09	< 0.001
	GAD-7 score		−0.02	−0.04 to 0.01	0.103		−0.03	−0.05 to −0.01	0.009

Cohort models used data from time-points one to three. RCT models used baseline data only. For valence, coefficients represent the number of hits for positive relative to negative words

^a^Confounders were age, gender, education level, negative life events and antidepressant use (current in the cohort and past in the RCT)

In the RCT, there was strong evidence that participants made more positive than negative hits (coef = 0.86, 95% CI = 0.63 to 1.09, *p* < 0.001 adjusted for confounders). However, there was only evidence that a higher GAD-7 score was associated with fewer hits after adjusting for confounders (coef=−0.03, 95% CI=−0.05 to −0.01, *p* = 0.009). In contrast to the cohort, there was no evidence for an interaction between word valence and GAD scores on the total number of hits, both before (interaction term *p* = 0.504) and after (interaction term *p* = 0.484) adjusting for the confounders. Despite this, when estimating associations between GAD scores and hits separately for positive and negative words, the association between GAD scores and total hits for positive words was larger (coef=−0.04, 95% CI=−0.06 to −0.01) than for negative words (coef=−0.02, 95% CI=−0.06 to 0.01).

## Discussion

We found evidence across two independent samples that, concurrently, better recall of socially rewarding information was associated with lower GAD scores. We found no evidence that recall of socially threatening information was associated with GAD scores. This suggests that decreased processing of socially rewarding information, rather than increased processing of socially threatening information, is a cognitive characteristic of GAD. Although the lagged models provided estimates in the same direction as in the concurrent models, we did not find evidence that recall of either socially rewarding or threatening information was associated with subsequent GAD scores. This suggests that changes in cognitive processing may not precede changes in GAD scores. In sensitivity analyses, we found evidence from the cohort study that the association between recall and GAD scores differed significantly according to word valence. Recall of socially rewarding information was more strongly associated with GAD scores than recall of socially threatening information. Although we did not replicate this evidence in the RCT, this does not contradict our overall conclusions, as the associations were in the same direction.

### Strengths and limitations

To our knowledge, this is the largest study of recall of social information and GAD symptoms. We replicated our findings using two independent samples (cohort vs. RCT), increasing the reliability and reproducibility of our results. The sample also covered the full range of GAD symptom severity. This increases the generalisability of the findings to individuals experiencing GAD symptoms in the community. Participants with the whole range of GAD symptoms were recruited from the same population, which should reduce the risk of selection bias compared with case-control studies where controls were recruited in a different way to patients with anxiety disorders.

The prospective design with repeated measures in the cohort study enabled us to take account of changes in recall and GAD scores over time. The multilevel linear models combined available data from all time-points, which should increase the statistical power and precision of the estimates [30]. The cohort had a high retention rate and there were no differences in the demographic characteristics and recall task performance of those who dropped out compared to those who completed all time-points. However, the data we included from both studies were observational, and there were only small changes in task performance and GAD scores over time, reducing the statistical power for the lagged analyses.

The response rates in both samples were low, which may reduce the generalisability of the results. However, this is unlikely to have biased our findings, as the selection of the participants was not based on recall performance. Another potential limitation is practice effects in the cohort sample. Although different sets of words were presented at each time-point, only recall at time-point one could be considered a surprise task, as participants may have expected the same task in the following time-points. However, we did not observe an improvement in overall task performance from time-point one to three in the cohort study, suggesting that practice effects were unlikely to have affected our findings. Additionally, in the sensitivity analyses, the tests of interaction were likely underpowered, which may explain why we did not replicate findings from the cohort study in the RCT. Additionally, although we adjusted for several potential confounders, it is still possible that residual confounding could have biased our results.

Another limitation of our study is that all participants had a history of depressive symptoms, as both samples were recruited based on this criteria. This may limit the generalisability of our findings to individuals with GAD symptoms but no history of depressive symptoms. Although our findings were similar to the associations with depressive symptoms found in other studies using the same cohort [21, 31], given the high comorbidity between depression and anxiety [32] and their shared genetic components [33], it remains unclear whether the observed recall patterns are specific to GAD or reflect common cognitive characteristics across mood and anxiety disorders. We therefore cannot conclude whether there are specific cognitive patterns that can distinguish between depression and GAD. To establish the specificity of cognitive patterns to generalised anxiety, future research could identify cognitive characteristics unique to individuals with GAD without comorbid depressive symptoms.

Finally, the relatively small changes in GAD scores over the six-week follow-up period may have limited our ability to detect lagged associations between recall biases and subsequent symptoms. The brief follow-up period and naturally occurring symptom fluctuations in a community sample may not provide sufficient variation in GAD scores to detect meaningful associations. Future longitudinal studies with longer follow-up periods, samples experiencing greater symptom variability, or populations undergoing treatment may be better positioned to examine whether recall biases predict subsequent changes in anxiety symptoms.

### Findings in context

Previous meta-analyses and case-control studies have found evidence for recalling less positive information in people with more severe anxiety across a range of anxiety disorders [10, 11][34–37]. In line with such findings, we found greater people recalling more socially rewarding information in people with fewer symptoms of GAD concurrently. However, our findings did not support cognitive theories of anxiety that predict selective retrieval of threatening information in people with anxiety [3, 4, 15]. We found no evidence for associations between recall of socially threatening words and GAD symptom scores. Hence, our findings suggest that generalised anxiety may be characterised by difficulty in remembering positive socially rewarding information but not better memory for threatening stimuli.

Given the role of executive functioning deficits in the aetiology and maintenance of GAD symptoms [38–40], it is possible that the recall biases found in this study were a result of poorer executive functioning. However, as associations differed according to word valence (they were only present for socially rewarding information), we would not expect this to be a result of differences in executive function. We explored this further in sensitivity analyses, demonstrating in the cohort sample that the association with overall task performance did differ according to word valence. Yet, findings from sensitivity analyses in the RCT sample were more consistent with the executive functioning explanation, as there was only evidence for an association between GAD symptom scores and overall recall, which did not differ significantly according to word valence. Despite this, associations were in the same direction as in the cohort sample. Further research is thus required to establish whether there are distinct contributions of executive functioning deficits and information processing biases in the recall of social information.

The finding that GAD symptoms were associated with poorer recall of socially rewarding information, but not enhanced recall of threatening information, differs from cognitive theories proposing that enhanced processing of threat-related information has a causal role in anxiety [2, 4]. Our results suggest that GAD may be characterised more by deficits in positive information processing than by enhanced negative information processing. One possible explanation is that cognitive resources in individuals with GAD are predominantly allocated to worry and threat monitoring [9], which may specifically interfere with the encoding and consolidation of positive social information whilst leaving negative information processing relatively intact. Future research could examine whether experimental manipulation of positive information processing leads to changes in GAD symptoms.

The lack of evidence for the lagged association between recall and subsequent GAD scores could suggest alternative mechanisms for the maintenance of GAD. There is strong evidence for the role of attentional and interpretive biases in anxiety [4–6]. This evidence is supported by two cognitive models of GAD, the metacognitive model [41] and the intolerance of uncertainty model (IUM; [42]). The metacognitive model proposes that individuals with GAD will have positive beliefs about worrying as a helpful coping strategy in the first instance, which means increased attention to threatening information forms part of the maintenance cycle of GAD [41]. The IUM suggests that individuals with GAD find uncertain or ambiguous situations stressful and upsetting and experience chronic worry in response to such situations [42]. Both models include a role for attentional and interpretive bias in maintaining GAD scores. However, we are not aware of any longitudinal research testing the impact of attentional and interpretive bias on subsequent GAD scores. Given that these models were conceptualised to describe both the development and maintenance of GAD, more longitudinal evidence is needed. Whilst our findings suggest that bias in recall of socially rewarding information is a potential marker of current GAD symptom scores, they highlight the need for further research. Exploring whether different information processing biases (i.e., attentional, interpretive and recall bias) have a causal role in GAD would improve our understanding of the cognitive aetiology of anxiety symptoms.

Overall, our findings indicate that biases in recall of socially rewarding information may be a marker of current GAD symptom severity among those who also have depressive symptoms, although they are unlikely to be a risk factor for subsequent GAD symptoms. In contrast, we found no evidence that recall of socially threatening information was associated with GAD symptom severity. We replicated these findings in two independent samples, with individuals who were and were not taking antidepressants. Future research should further explore the cognitive aetiology underlying the maintenance of GAD symptoms using longitudinal studies with large samples recruited from the general population.

## Supplementary Information

Supplementary Material 1.

## Data Availability

No datasets were generated or analysed during the current study.

## Abbreviations

CIS-R: Clinical interview cchedule - revised
GAD: Generalised anxiety disorder
GAD-7: Generalised anxiety disorder assessment - 7-item version
GP: General practice
ICD-10: International classification of diseases - tenth edition
PANDA: The prescribing antidepressAnts research programme
RCT: Randomised controlled trial
