# Supplementary material for: Variation in the recall of socially rewarding information and symptoms of generalised anxiety: evidence from two cohorts
Source: BMC Psychiatry. 2025 Oct 21;25:1009. doi: 10.1186/s12888-025-07402-1 (PMC12539046; doi:10.1186/s12888-025-07402-1)
Supplement: Supplementary file 1 — Supplementary Material 1. [file 12888_2025_7402_MOESM1_ESM.docx]

**Supplementary Materials**

***Table S1.*** Demographics and recall performance among participants who dropped out (i.e., completed three or fewer time-points) compared to those who completed all four time-points in the cohort study.

|  | | **Completed  1-3 time-points**  (N = 128) | **Completed  all time-points**  (N = 430) |
| --- | --- | --- | --- |
|  | | N (%) | |
| Age group | Under 50 | 70 (53%) | 201 (48%) |
|  | 50 or older | 62 (48%) | 226 (52%) |
| Gender | Male | 41 (31%) | 138 (32%) |
|  | Female | 91 (69%) | 288 (68%) |
| Ethnicity | White | 119 (90%) | 415 (97%) |
|  | Non-white | 13 (10%) | 11 (3%) |
| Education level | GCSE or below | 62 (47%) | 155 (36%) |
|  | A level or above | 69 (53%) | 271 (64%) |
| Marital status | Married or cohabiting | 53 (40%) | 225 (53%) |
|  | Single, separated, divorced or widowed | 79 (60%) | 201 (47%) |
| Employment status | Employed | 65 (49%) | 233 (55%) |
|  | Unemployed | 35 (27%) | 89 (20%) |
|  | Student | 7 (6%) | 3 (1%) |
|  | Retired/full-time carer | 24 (18%) | 101 (24%) |
| Negative life events | None | 37 (28%) | 282 (66%) |
|  | One or more | 95 (72%) | 144 (34%) |
| Current antidepressant use | | 116 (88%) | 373 (88%) |
| GAD diagnosis |  | 62 (47%) | 182 (43%) |
| Depression diagnosis |  | 66 (50%) | 193 (45%) |
|  |  | Mean (standard deviation) | |
| Age (continuous) |  | 46.67 (12.63) | 48.94 (12.57) |
| Positive hits |  | 2.43 (1.96) | 2.38 (1.76) |
| Negative hits |  | 1.80 (1.50) | 1.61 (1.37) |

*Note.* Recall task performance was missing for some participants. Data on positive hits were available for 111 participants who completed 1-3 time-points and 418 who completed all time-points. Data on negative hits were available for 109 participants who completed 1-3 time-points and 411 who completed all time-points.
